# THE BENEFITS OF CREATIVE ACTIVITIES FOR DIVERSE GROUPS OF OLDER ADULTS

**DOI:** 10.1093/geroni/igae098.2236

**Published:** 2024-12-31

**Authors:** Carolyn Adams-Price, Justine McGovern

**Affiliations:** Chair; Discussant

## Abstract

Participation in creative activities is said to be beneficial for older adults and is often suggested for healthy cognitively intact and cognitively impaired populations. The purpose of this symposium will be to explore the benefits of different kinds of creative programs and interventions offered to diverse groups of older adults of different ability levels. The first paper in this symposium will discuss theory and the psychosocial context of creativity in older adults. The observed and hypothetical benefits will also be discussed from different frameworks, including life-span developmental psychology and dementia studies. LaVona Traywick will present the physical and emotional benefits of her dance program with older adults with Parkinson’s disease. Jennifer Dorris will discuss her experience incorporating music into her activities with older Down’s syndrome patients. Justine McGovern will be a discussant for this session, tying together the different presentations.

